# Gastrointestinal Stromal Tumors—A Mini Review

**DOI:** 10.3390/jpm11080694

**Published:** 2021-07-22

**Authors:** Gina Gheorghe, Nicolae Bacalbasa, Gabriela Ceobanu, Madalina Ilie, Valentin Enache, Gabriel Constantinescu, Simona Bungau, Camelia Cristina Diaconu

**Affiliations:** 1 Clinical Emergency Hospital of Bucharest, Department of Gastroenterology, University of Medicine and Pharmacy “Carol Davila“, 050474 Bucharest, Romania; gheorghe_gina2000@yahoo.com (G.G.); drmadalina@gmail.com (M.I.); 2 Department of Visceral Surgery, “Fundeni” Clinical Institute, University of Medicine and Pharmacy “Carol Davila“, 050474 Bucharest, Romania; nicolae_bacalbasa@yahoo.ro; 3 “Sfanta Maria” Clinical Hospital, Department of Internal Medicine and Rheumatology, 011172 Bucharest, Romania; gabriela.ceobanu@ymail.com; 4 Clinical Emergency Hospital of Bucharest, Department of Anatomical Pathology, 050474 Bucharest, Romania; valienache@rocketmail.com; 5 Department of Pharmacy, Faculty of Medicine and Pharmacy, University of Oradea, 410028 Oradea, Romania; simonabungau@gmail.com; 6 Clinical Emergency Hospital of Bucharest, Department of Internal Medicine, University of Medicine and Pharmacy “Carol Davila”, 050474 Bucharest, Romania; drcameliadiaconu@gmail.com

**Keywords:** GIST, c-Kit, CD-117, interstitial cell of Cajal

## Abstract

Gastrointestinal stromal tumors (GISTs) are the most common mesenchymal neoplasms of the gastrointestinal tract. They are potentially malignant, and have an unpredictable evolution. The origin of these tumors is in the interstitial cells of Cajal, which are cells that are interposed between the intramural neurons and the smooth muscle cells of the digestive tract. GISTs are characterized by mutations in the gene c-Kit, but also other mutations, such as those of the platelet-derived growth factor receptor alpha. The most common locations of these tumors are the stomach and small intestine, although they can occur at any level of the digestive tract and occasionally in the omentum, mesentery and peritoneum. Most cases of GISTs are sporadic, and about 5% of cases are part of family genetic syndromes. The correct diagnosis of GIST is determined by histopathological examination and immunohistochemistry. According to histopathology, there are three main types of GISTs: spindle cell type, epithelioid type and mixed type. The therapeutic management of GIST includes surgery, endoscopic treatment and chemotherapy. The prognosis of patients with GIST varies depending on a number of factors, such as risk category, GIST stage, treatment applied and recurrence after treatment.

## 1. Introduction

Gastrointestinal stromal tumors (GISTs) are non-epithelial neoplasms, involving the gastrointestinal tract. These mesenchymal tumors account for only about 1% of all primary malignant tumors of the gastrointestinal tract [1]. The worldwide incidence of GISTs is estimated to be 7–15 cases per 1 million people per year [2,3]. The incidence varies depending on the geographical area. Thus, in Western countries, the incidence of GISTs is estimated to be 10–15 cases per million people per year, while in Asia it is estimated to be 16–20 cases per million people per year [2]. In the United States, approximately 6000 new cases of GISTs are diagnosed annually [4]. However, studies that have analyzed data that were obtained from necropsy examination suggest that the real incidence of these mesenchymal tumors is underestimated. Necroptic studies incidentally revealed a significant number of patients with GIST, with dimensions up to 1 cm [5,6]. These tumors are most commonly diagnosed in individuals aged between 50 and 70 years old. In terms of gender distribution, the ratio of men to women is approximately equal [6].

The survival rate of patients with GIST varies depending on the following factors: risk category or GIST stage, treatment applied and recurrence after treatment. Thus, patients with localized GISTs have a 5-year life expectancy of 93%, while patients with locally advanced GISTs have a 5-year survival rate of 80%, and those with metastatic GISTs of 55% [7].

GISTs were originally described in 1980 as smooth muscle tumors, but the development of immunohistochemical and molecular diagnostic methods have led to the definition of GISTs as a distinct category [8]. An important finding was the identification of CD117 antigen expression in nearly all GISTs, thus differentiating them from leiomyosarcomas, leiomyomas and other spindle cell tumors of the gastrointestinal tract, which are CD117 negative [9]. The interstitial cells of Cajal (ICCs) expressing c-Kit (CD117), a type III tyrosine kinase receptor for stem cell growth factor, were found to be the source of GISTs. These cells, sometimes referred to as gastrointestinal pacemaker cells, have particular immunophenotypic and ultrastructural characteristics that make them generate slow electric waves [10]. The role of ICCs lies in the regulation of peristalsis [11]. The immunohistochemical examination reveals that 95% of GISTs are positive for KIT (CD117), and 70% are positive for CD34 [10,12,13]. Patients with GISTs can associate mutations in the platelet-derived growth factor receptor alpha (PDGFRA), succinate dehydrogenase complex or the BRAF; rarely, they can associate mutations in the RAS family genes [12,13]. GISTs are most commonly found in the stomach (60%) and small intestine (20–30%), but they may appear at any level of the digestive tract and occasionally in the omentum, mesentery and peritoneum [12,13]. Tumors located outside the gastrointestinal tract are rare, and it is believed that they originate in the ICCs, in which accidentally disperse during embryogenesis [13,14].

## 2. Classification of GIST

### 2.1. Etiological Classification of GISTs

Most cases of GIST are sporadic, and about 5% belong to the family of genetic syndromes, such as:

- Carney–Stratakis syndrome (dyad).
- Carney triad syndrome.
- Family Neurofibromatosis type 1 (NF1).
- Primary familial GIST syndrome [15,16].

Currently, sporadic cases of GISTs cannot be differentiated from familial cases of GISTs, from a phenotypic, histological or molecular point of view [17,18].

Carney–Stratakis syndrome or Carney–Stratakis dyad is diagnosed in children or young adults at an average age of 19–21 years [19]. These patients usually have associate GISTs and paragangliomas [19]. Carney triad is diagnosed in young women with GISTs, pulmonary chondromas and paragangliomas [19].

Carney–Stratakis syndrome and Carney triad are GIST syndromes diagnosed in pediatric, adolescent and young adult patients [19].

Recently, it has been shown that Carney–Stratakis syndrome and Carney triad are characterized by mutations and methylation changes of succinate dehydrogenase (SDH) subunit genes, which in turn lead to global SDH deficiency. In contrast, in the case of NF1 and primary familial GIST syndrome, patients remain SDH competent. In SDH-deficiency syndromes, the recommendations for treatment and monitoring are different [16]. In most cases, these patients are part of clinical trials, or their treatment takes place in tertiary care centers [20]. There are data suggesting that surgical resection may not be beneficial for some patients with non-KIT/PDGFRA-mutated tumors [20]. Furthermore, SDH-deficient GISTs are frequently resistant to tyrosine kinase inhibitors (TKIs), normally used in patients with advanced GISTs and KIT/PDGFRA mutation. This can be explained by the absence of gain-of-function tyrosine kinase mutation. However, although limited efficiency of these therapeutic agents is demonstrated, some patients with SDH-deficient GISTs may benefit from this treatment [20]. In terms of the surveillance of patients with SDH-deficient GISTs, there are no generally accepted recommendations. In addition, asymptomatic individuals carrying SDHx mutation must be monitored, due to their predisposition to develop neoplastic disorders [20].

GISTs associated with NF1 syndrome are localized in the small intestine in >70% of cases [16,17]. These are usually multifocal tumors, and have low mitotic rates [17,18]. Unlike sporadic GISTs, in these cases, mutations in the PDGFRA and KIT genes are rare [17,18].

The primary familial GIST syndrome is characterized by the predisposition to an early development of multiple tumors, located in the stomach or small intestine [21]. Patients with germline mutations in KIT genes can have associate paragangliomas, dysphagia or skin hyperpigmentation, and patients with mutations in PDGFRA genes associate inflammatory fibroid polyps or intestinal fibromatosis [21,22].

In accordance with the location of the origin cells, the tumor appears at the sub-epithelial level.

### 2.2. Histological Classification of GISTs

Macroscopically, GISTs are white in color, well-defined, not encapsulated and have a firm consistency [23]. The section surface may be homogeneous, seen mostly in small-size GISTs, or heterogeneous, with areas of hemorrhage and necrosis in larger tumors. In small tumors, the coating mucosa remains unchanged (appearing normal), but in large, more aggressive tumors, it may ulcerate. Microscopically, GISTs may have a moderate or high cellularity and can be divided into three main types [23,24]:

- Spindle cell type (70%);
- Epithelioid type (20%);
- Mixed type (10%).

GISTs of the spindle cell type are composed of eosinophil cells that have a slightly paler cytoplasm compared to that of leiomyoma. Nuclei are usually uniform, but juxtanuclear cytoplasmic vacuoles and nuclear palisading may be visible. These cells are arranged in short fascicles or whorls [24].

Epithelioid GISTs are composed of rounded epithelioid cells that have a clear, eosinophilic cytoplasm and round or oval nuclei. Tumors of this type are located mostly in the stomach, and more often their KIT expression is negative and can harbor PDGFRA [25,26].

Mixed type GISTs are tumors that contain both types of cells, fusiform and epithelioid.

### 2.3. Immunohistochemical Classification of GISTs

Immunohistochemically, the markers that may contribute to the differentiation of GISTs from other subepithelial tumors in the gastrointestinal tract are:

- KIT (CD117);
- DOG-1 (discovered on GIST-1) and protein kinase C theta (PKC-theta);
- Other markers: CD34, smooth muscle actin, S-100 protein, desmin and keratin [27,28].

### 2.4. Molecular Classification of GISTs

From a molecular point of view, the mutations found in GIST are as follows:

- In total, 75% of cases harbor KIT mutations (usually in exons 11, 9 and rarely in exons 13, 17, 14 and 18) [29,30].
- A total of 10% of cases harbor PDGFRA mutations (exon 18: D842V with important resistance to imatinib and non-D842V with sensitivity to imatinib; exon 12, and rarely exons 14 and 10) [31,32].
- In total, 10–15% are KIT/PDGFRA wild-type [33,34]:
  - One third (20–40%) have SDH deficiency: SDHx mutations or SDHC promoter hypermethylation [33,34].
  - About 13% of cases have BRAF (V600E) or NF1 mutations [34].
  - Rarer events: fusions on ETV6-NTRK3, FGFR1 fusion or point mutations and FGF4 duplication [33,34,35].

GISTs that do not present KIT/PDGFRA mutations form the group named KIT/PDGFRA wild-type (WT GIST). Currently, detailed molecular analysis has shown that this group is heterogeneous and has several mutations (Table 1) [33,34].

## 3. Clinical Manifestations of GISTs

Clinical manifestations vary according to tumor location and size. Small size GISTs are usually asymptomatic and are diagnosed incidentally during an endoscopic exploration, on radiological imaging for a different purpose, or during surgery [36]. In the absence of complications, such as upper digestive hemorrhage/hemoperitoneum, tumor perforation, intestinal obstruction or obstructive jaundice, the symptoms are nonspecific (early satiety, anemia, abdominal pain, swelling) [37,38]. The clinical onset of GISTs is described in Table 2 [36,37,38].

The most common manifestation is gastrointestinal bleeding, which may be accompanied by anemia, melena or hematemesis. Large tumors may cause abdominal distention, obstruction of the gastrointestinal lumen (tumors with endophytic growth) or compression of the gastrointestinal tract (GISTs with exophytic growth). Dysphagia is the first specific symptom encountered in esophageal GISTs. Furthermore, in advanced stages, metastases can occur. It was reported that up to 50% of patients with GISTs may develop metastases. Moreover, because of the diagnosis delay, a significant number of patients present with metastases at the time of diagnosis [39]. Most often, metastases are localized in the liver (65%) and peritoneum (21%). Rarely, GISTs can metastasize in bones, lungs and lymph nodes [23].

## 4. Diagnostic Work-Up

**Figure 1:**
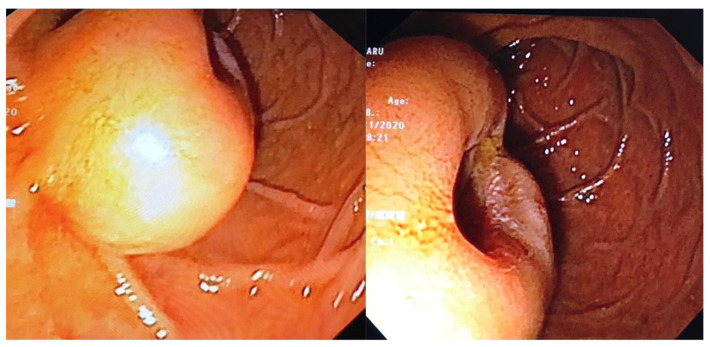
Macroscopic aspect of a duodenal GIST—round-oval shaped tumor, well-delimited, with dimensions of approximately 5 mm and excavated center, covered by normal mucosa (endoscopic examination). From the collection of Dr. Madalina Ilie.

**Figure 2:**
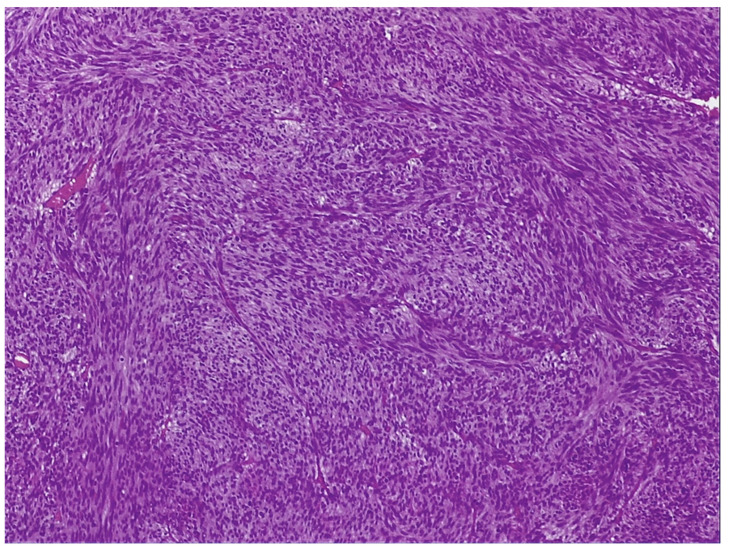
Histological aspect of a spindle cell type in GIST-Bland spindle cells with eosinophilic cytoplasm in a syncytial pattern, elongated nuclei with inconspicuous nucleoli (Hematoxylin-Eosin, 100×). From the collection of Dr. Valentin Enache.

**Figure 3:**
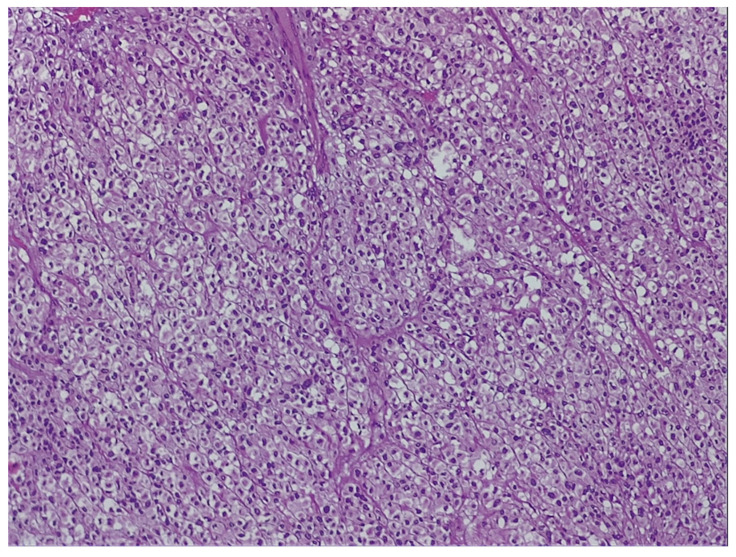
Histological aspects of an epithelioid type of GIST-Round cells with clear to eosinophilic cytoplasm in sheets or nests (Hematoxylin-Eosin, 100×). From the collection of Dr. Valentin Enache.

**Figure 4:**
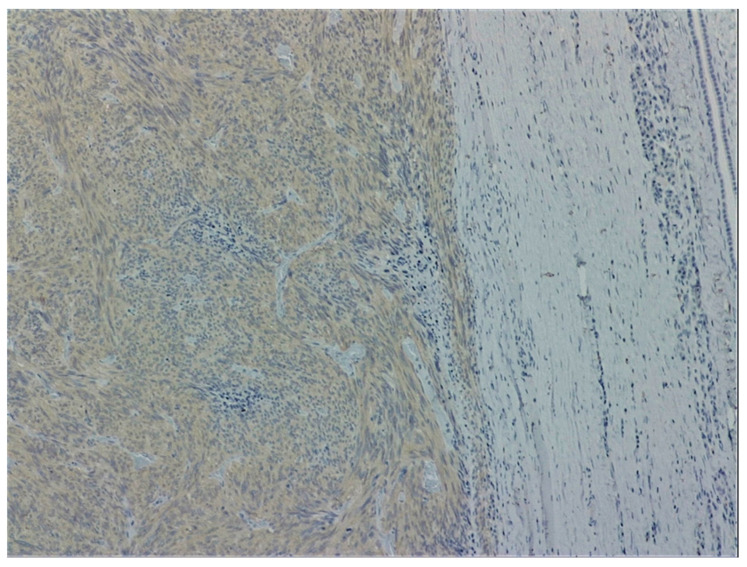
Histological aspects of spindle cell type of a GIST. CD117 diffuse cytoplasmic staining in tumor cells (CD117, 100×). From the collection of Dr. Valentin Enache.

**Figure 5:**
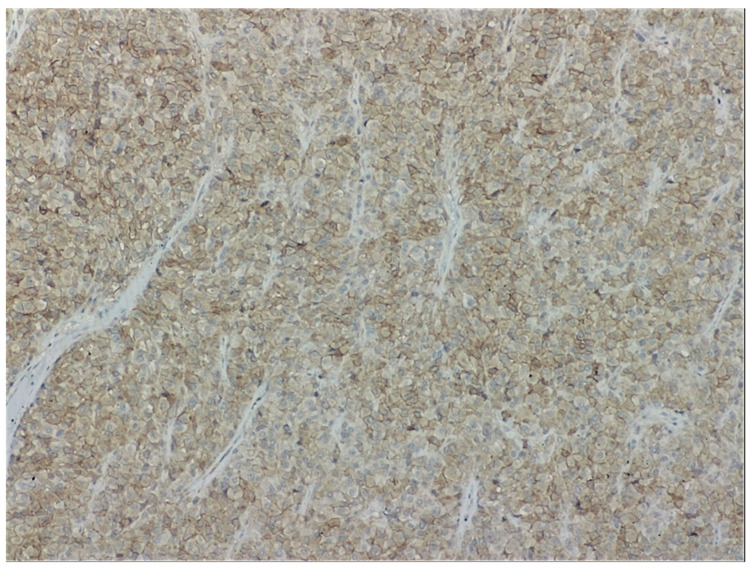
Histological aspects of an epithelioid type of GIST-DOG1 positive. Membranous and cytoplasmic staining in tumor cells (DOG1, 100×). From the collection of Dr. Valentin Enache.

a. Endoscopic examination has an essential role in definitive diagnosis because it allows the direct visualization of the tumor, with the possibility of biopsies for pathological examination (Figure 1). Both GISTs and leiomyomas may emerge as tumors with smooth margins located in the submucosa, with a normal mucosa cover that bulges into the lumen of the digestive tract. In some cases, a central ulceration may be seen.
b. Endoscopic ultrasonography (EUS) permits the assessment of the invasion within the gastrointestinal wall and identification of the digestive tract layer as an origin for the GIST. Thus, most often, GISTs originate in the muscularis propria, and small lesions may also originate from the muscularis mucosa [40]. Upon EUS, GISTs appear as a hypoechoic, homogeneous tumor, with clearly defined edges, rarely irregular and sometimes with associated ulcers. EUS also enables both guided biopsies and GIST differentiation to other submucosal tumors [40].
c. Contrast-enhanced computed tomography (CT) is the imaging method of choice to identify and describe the neoplasms, as well as to assess their extension and the presence of metastatic disease. Thus, CT allows the identification of metastases, which are most commonly located in the liver, omentum and peritoneal cavity. It also allows differential diagnosis, assessment of response to treatment and identification of tumor recurrence [41].
d. Abdominal ultrasound, magnetic resonance imaging (MRI) and positron emission tomography (PET) are also useful in the evaluation of GISTs and in the detection of metastases. Although MRI has a diagnostic performance comparable to that of CT, CT scan remains the preferred initial imaging method used for staging the disease. There are some cases in which MRI may be a better imaging option, such as in GISTs found in specific locations (e.g., the rectum) or in evaluating the anatomical extension of surgery [42].
e. The definitive diagnosis is histopathological. Biological samples may be obtained during endoscopic exploration, laparoscopic excision or laparotomy. In the case of metastases, the samples for histopathological diagnosis can also be obtained by biopsy of the metastases [43]. Depending on the tumor cell appearance after hematoxylin and eosin staining, three morphological types have been identified: spindle cell type, epithelioid type and mixed type [43,44]. Figure 2 shows the histological aspects of the spindle cell type in GISTs, and Figure 3 presents the histological aspects of the epithelioid type in GISTs.
f. Immunohistochemistry is essential for the diagnosis of GISTs. In over 95% of cases, GISTs are positive for CD117/c-Kit. Other markers used for the diagnosis of GISTs are DOG1, CD34, S-100 protein, SMA and Ki67 (Figure 4 and Figure 5) [45,46].

**Figure 6:**
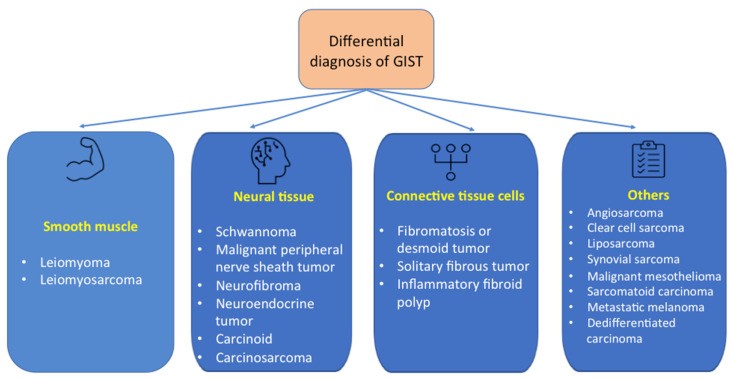
Differential diagnosis of GISTs.

The differential diagnosis is made with other sub-epithelial tumors, as listed in Figure 6 [46,47,48].

Thus, differential diagnosis is made with tumors that originate in smooth muscles (leiomyoma, leiomyosarcoma), neural tissue (schwannoma, malignant peripheral nerve sheath tumor, neurofibroma, neuroendocrine tumor, carcinoid, carcinosarcoma), connective tissue cells (fibromatosis or desmoid tumor, solitary fibrous tumor, inflammatory fibroid polyp) and also with other tumors (angiosarcoma, clear cell sarcoma, liposarcoma, synovial sarcoma, malignant mesothelioma, sarcomatoid carcinoma, metastatic melanoma or dedifferentiated carcinoma) [46,47,48]. To differentiate between these types of neoplasms, histopathology, immunohistochemistry and genetics are useful. For example, there are three types of leiomyomas: intramural leiomyoma, leiomyoma of the muscular mucosa and Mullerian type leiomyoma. Intramural leiomyoma usually occurs at the esophageal level in young adults. Histologically, this tumor is characterized by the identification of eosinophilic spindle cells, which are positive for smooth muscle markers, but are negative for KIT or anoctamin 1 [46,47,48]. Leiomyoma of the muscular mucosa is usually identified in the large intestine, and in older adults has histopathological features similar to intramural leiomyoma [46,47,48]. Mullerian type leiomyoma can be identified in the colon and also in the abdominal cavity, and is characterized by its positivity for estrogen and progesterone receptor [46]. Leiomyosarcoma develops more frequently in the colon. These tumors have similar characteristics to leiomyoma, exhibiting additional mitotic activity and nuclear atypia [46,48]. Schwannoma usually appears in older adults, being characterized by spindle cells arranged in microtrabeculae or microfascicles. Immunohistochemically, these cells are negative for KIT and anoctamin 1, but are positive for S100 protein and glial fibrillary acidic protein (GFAP) [46,48]. Synovial sarcoma is a spindle cell tumor negative for KIT, but positive for keratin component [45]. Dedifferentiated liposarcoma is a spindle cell tumor which is also negative for KIT and anoctamin 1, but with nuclear positivity for mouse double minute 2 protein (MDM2) [46].

## 5. Risk Stratification

Risk stratification of GISTs attempts to assess the risk of a poor outcome and to identify patients who may benefit from adjuvant therapy. The evolution of GISTs is highly complex, making the prediction of their malignant potential extremely difficult [49]. Since all GISTs can be considered malignant, multiple consensus criteria have been developed over the years, allowing the stratification of GISTs based on the risk of metastasis or recurrence. Factors such as primary tumor size or location, mitotic activity, as well as rupture of the tumor either prior to or during surgery, failure to obtain clear margins during surgery, and deletions in KIT exon 11 are known to contribute to the malignant potential of GISTs. Clinically, the classification scores by Fletcher et al. and Miettinen and Lasota are the most widely accepted [10,50,51]. Fletcher et al. classify the risk of aggressive evolution in four classes, depending on tumor size and mitotic rate:

- Very low risk: tumoral size <2 cm; mitotic count <5/50 high-power field (HPF).
- Low risk: tumoral size 2–5 cm; mitotic count <5/50 HPF.
- Intermediate risk: tumoral size <5 cm and mitotic count 6–10/50 HPF, or tumoral size 5–10 cm and mitotic count <5/50 HPF.
- High risk: tumoral size >5 cm and mitotic count >5/50 HPF, or tumoral size >10 cm and any mitotic rate, or any tumoral size and mitotic rate >10/50 HPF [10].

In 2006, Miettinen et al. proposed another classification score for the risk of aggressive evolution of GISTs. This score uses, in addition to tumor size and mitotic rate, the location of the primary tumor [51]. These authors demonstrated that the gastric localization of GISTs is associated with a better prognosis, compared to GISTs that are localized in the small intestine or rectum [51].

## 6. Treatment of GIST

The standard treatment of localized GISTs is surgery. Both the tumor and its pseudocapsule should be removed to yield an adequate surgical margin, as the goal in primary GISTs is complete removal (R0). Due to the fact that GISTs rarely metastasize to lymph nodes, regional lymph node resection is not required [52]. The presence of metastases does not represent a contraindication for surgery of the primary tumor.

Other treatment methods for non-metastatic GISTs are endoscopic techniques, such as enucleation, submucosal dissection, submucosal excavation, band ligation, full-thickness resection, submucosal tunneling endoscopic resection and laparoscopic and endoscopic cooperative surgery [53,54,55].

Endo-sonographically, GISTs are classified into four subtypes, depending on their localization reported to the muscularis propria:

- Type I: a tumor protruding into the digestive lumen while being narrowly connected to the muscularis propria.
- Type II: a tumor protruding into the digestive lumen while being widely connected to the muscularis propria.
- Type III: a tumor that is centrally localized on the gastric wall.
- Type IV: a tumor protruding into the serosa of the gastric wall [53,54,55].

Endoscopic enucleation can be used for type I and possibly for type II GISTs. Types III and IV may benefit from the following endoscopic treatment techniques: submucosal dissection, submucosal excavation, full-gross resection, submucosal tunneling resection and laparoscopic and endoscopic cooperative surgery [55].

Gastric GISTs with dimensions ≤4 cm can benefit from safe endoscopic resections. Gastric GISTs with dimensions >4 cm have a risk of recurrence or even metastasis, and may require adjuvant therapy with tyrosine kinase inhibitors (TKIs), or even the combination of an endoscopic and surgical technique [56].

About 85% of GISTs have mutations in KIT or PDGFRA receptors, which explains the favorable response to TKIs. However, 10–15% of patients with GISTs are part of a heterogeneous group, KIT/PDGFRA wild-type [54]. Half of them have been identified to be SDH deficient or BRAF/RAS/NF1 mutated, and the other half with quadruple WT GISTs have a greater molecular heterogeneity. For this group of patients, the therapeutic possibilities are very limited, as they usually present with resistance to TKIs [33,57].

A special category is represented by SDH-deficient tumors, which have a high rate of primary resistance to TKIs but a slow evolution. The therapeutic management of these patients is not yet clearly established. They usually do not respond to imatinib treatment, but may have a response to sunitinib or regorafenib, and may be candidates for various clinical trials [58,59].

In the pre-TKI era, the prognosis of patients with metastatic or unresectable GISTs was very poor. The first TKI approved by the Food and Drug Administration was imatinib mesylate, a first-line treatment for unresectable or metastatic GISTs and used as an adjuvant or neoadjuvant therapy. This was followed by sunitinib, a second-line TKI and finally regorafenib, a third-line TKI [60]. Adjuvant imatinib therapy should be considered a standard treatment in all patients who underwent resection of a primary GIST and have a significant risk of recurrence [61].

Patients who undergo a complete resection of the tumor, with negative margins and without tumor rupture, may still develop metastases or recurrence of the tumor. Therefore, patients with a GIST should be ensured access to a multidisciplinary team through early referral to a medical oncologist at a sarcoma reference center. Selection of patients eligible for adjuvant therapy should be based on risk assessment. Thus, patients with advanced disease require an initial assessment of tumor mutational status for targeted chemotherapy. Clinical responses to TKI are correlated with tumor genotype [60,61,62].

Neoadjuvant imatinib should be considered in patients with large tumors, in whom immediate resection is not possible (e.g., total gastrectomy) [60,61,62]. Currently, imatinib can be used for patients with KIT mutations and most PDGFRA mutations (except PDGFRA D842V) [60,61,62]. In addition, there are studies suggesting that the real percentage of patients with KIT/PDGFRA wild-type is lower than was considered. These studies explain this by genetic testing errors and missing KIT/PDGFRA mutations. Under these conditions, these authors suggested the usefulness of imatinib, even in patients with KIT/PDGFRA wild-type group [57].

In patients with unknown KIT mutational status, an alternative second-line treatment is sunitinib [62]. The presence of PDGFRA D842V mutation confers significant resistance to imatinib [62,63]. Thus, avapritinib is recommended in patients with symptomatic or rapidly progressive disease and PDGFRA D842V mutation [62,63]. However, if this mutation is identified but the patient is asymptomatic or has a form of indolent disease, an observation period prior to initiating avapritinib is recommended because of treatment toxicity and potential cognitive impairment. Other therapeutic alternatives for patients with PDGFRA D842V mutation are ripretinib or dasatinib (with limited data) [64,65]. The INVICTUS study demonstrates the improved survival rate of patients with advanced GISTs, through the use of ripretinib as a four-line TKI [64]. This study included 129 patients with advanced GISTs, with 10 of them presenting wild-type KIT and PDGFRA mutational status. Ripretinib led to an improvement in median progression-free survival in all patients included in the study and has a good safety profile [64]. In 2020, the United States Food and Drug Administration (FDA) has approved the use of ripretinib in patients with advanced GISTs, resistant to three or more TKIs, including imatinib [64]. Dasatinib is an inhibitor of KIT, PDGFR and the protooncogene tyrosine-protein kinase Src (SRC) [65]. SRC is expressed in GISTs, but the pathogenic role is not completely elucidated [65]. The results of the studies show that dasatinib can be used in some patients with GISTs who are resistant to imatinib [65]. However, future studies are needed to establish the efficacy and safety profile of this drug. Currently, it is not approved for the treatment of these diseases [65].

Table 3 shows the treatment with TKIs according to mutational status.

### Despite All T

Despite therapeutic advances, nearly one third of patients with GISTs, including those with extended TKI therapy, will experience a recurrence [55,66]. In these patients, careful management and follow-up is essential.

The follow-up schedule differs depending on the risk of recurrence. For example, in high-risk patients, there is a risk of recurrence in 1–3 years after the end of adjuvant therapy. In low-risk patients, the risk of recurrence is lower, and the time to recurrence is longer [67,68].

Patients at very low risk may not require postoperative follow-up, although the risk of recurrence is not zero. In low-risk patients, a CT scan examination is recommended every 6 months for 5 years. Intermediate–high risk patients require postoperative follow-up by CT examination at 3–4 months in the first 3 years, then at 6 months for 5 years, then annually [66,67,68]. There is a consensus that abdominal ultrasonography can replace CT evaluation once a year [68]. In patients that are undergoing TKI therapy, PET-CT is more sensitive for assessing treatment response, treatment resistance or tumor recurrence [69].

## 7. Conclusions

GISTs are mesenchymal tumors that may occur sporadically, or as part of a familial genetic syndrome. The natural evolution of these tumors is variable. Negative prognostic factors are young age, higher tumor size, increased mitotic index, aneuploidy and tumor location. Gastric tumors have a better prognosis than those localized in the intestinum [70,71]. For a positive diagnosis and differentiation between GISTs and other tumors with the same localization, histopathological and immunohistochemical tests are necessary [47,72]. These tests are very important, as the therapeutical management is different depending on the histology of the tumors. Patients with advanced GISTs require the assessment of mutational status for personalized chemotherapy with TKIs. The use of TKIs has led to an improvement in survival rate and quality of life of these patients. Proper treatment can improve the prognosis of patients and the epidemiological indicators, such as morbidity and mortality.

## Figures and Tables

**Table 1:** Classification and characteristics of KIT/PDGFRA wild-type (adapted after [33,34]).

Molecular Mutation	Characteristics
SDH deficiency (SDHx mutations or SDHC promoter hypermethylation)	Age: young age
	Sex predilection: female
	Localization: stomach
	Morphology: mixed epithelioid and spindle cell
	Over-expression of the IGF1R (insulin growth factor 1 receptor)
	Behavior: indolent evolution
2. BRAF V600E mutation	Localization: small intestine
	Prognosis more favorable
3. NF1 mutation	Age: adult
	Sex predilection: female
	Localization: multifocal localization; frequent non-gastric site
4. Quadruple WT-GIST	Molecular heterogeneity:
	- Gene fusion such as ETV6-NTRK3, FGFR1–HOOK3, FGFR1–TACC1, KIT–PDGFRA, MARK2-PPFIA1, SPRED2-NELFCD
	- somatic mutations such as TP53, MEN1, MAX, CHD4, FGFR1, CTDNN2, CBL, ARID1A, BCOR, APC

**Table 2:** Clinical manifestations of patients with GISTs.

Clinical Manifestations	Frequency
Overt or occult gastrointestinal bleeding	28%—small intestine
	50%—stomach
Incidental finding (asymptomatic)	13–18%
Abdominal pain/discomfort	8–17%
Acute abdomen	2–14%
Asymptomatic abdominal mass	5%

**Table 3:** TKIs and mutational status of GIST.

Mutational Status	TKI
KIT mutations and most PDGFRA mutation (except PDGFRA D842V)	- First line: imatinib - Second line: sunitinib - Third line: regorafenib
2. PDGFRA D842V mutation	- Avapritinib - Other therapeutic alternatives: ripretinib, dasatinib
3. SDH-deficient GIST	- Sunitinib - Regorafenib - Candidates for various clinical trials

## Data Availability

Not applicable.
